# Prevalence of extraintestinal manifestations in Korean inflammatory bowel disease patients

**DOI:** 10.1371/journal.pone.0200363

**Published:** 2018-07-10

**Authors:** Bo Ram Yang, Nam-Kyong Choi, Mi-Sook Kim, Jaeyoung Chun, Sang Hyun Joo, Hyesung Kim, Joongyub Lee

**Affiliations:** 1 Medical Research Collaborating Center, Seoul National University Hospital, Seoul, Republic of Korea; 2 Department of Health Convergence, Ewha Womans University, Seoul, Republic of Korea; 3 Department of Preventive Medicine, Seoul National University College of Medicine, Seoul, Republic of Korea; 4 Department of Internal Medicine and Liver Research Institute, Seoul National University College of Medicine, Seoul, Republic of Korea; 5 Division of Rheumatology, Department of Internal Medicine, Seoul National University Hospital, Seoul, Republic of Korea; 6 Medical Affairs, Janssen Korea, Seoul, Republic of Korea; 7 School of Medicine, Inha University, Incheon, Republic of Korea; 8 Department of Prevention and Management, Inha University Hospital, Incheon, Republic of Korea; Institute of Subtropical Agriculture, Chinese Academy of Sciences, CHINA

## Abstract

**Background:**

The prevalence of inflammatory bowel disease (IBD) in South Korea is increasing. Although extraintestinal manifestations (EIMs) are an important factor in the clinical outcomes of IBD patients, EIMs have not yet been investigated in Korea. Thus, we conducted a cross-sectional study to assess the prevalence of EIMs in Korean IBD patients.

**Methods:**

The 2014 claims data from the National Health Insurance System (NHIS) of Korea were used. IBD patients were identified by codes for Crohn disease (CD) and ulcerative colitis (UC) in the NHIS registration system for rare or intractable diseases. International Classification of Diseases, Tenth Edition codes were used to identify EIM cases. To estimate the prevalence of EIMs in the general population of Korea, we used national sample data. Standardized prevalence ratios (SPRs) were calculated to compare the prevalence rates of EIMs among IBD patients to those among the general population of Korea.

**Results:**

A total of 13,925 CD patients and 29,356 UC patients were identified. CD and UC patients were different in terms of demographics and utilization of medication. Among the 17 EIMs investigated, pyoderma gangrenosum, osteomalacia, Sweet syndrome, and scleritis were observed in very few patients. The SPRs were greater than 1 for all EIMs. Aphthous stomatitis, rheumatoid arthritis, and osteoporosis were highly prevalent in both CD and UC patients, but the SPRs of the EIMs were not high.

**Conclusion:**

The study confirmed that EIMs are more prevalent among IBD patients than among the general population of Korea. The prevalence of EIMs in IBD patients suggests the need for greater attention and effort in clinical practice.

## Introduction

Inflammatory bowel disease (IBD) includes Crohn disease (CD) and ulcerative colitis (UC). IBD is a systemic inflammatory disease that can affect all parts of the body, meaning that it can have extraintestinal manifestations (EIMs). EIMs occur most frequently in the joints, skin, the hepatobiliary system, and the eye [1–4]. It has been reported that 6%-47% of IBD patients experience EIMs, with considerable variation across studies [5–8]. Approximately a quarter of EIMs are first identified before the onset of IBD, and the remaining 75% are diagnosed after the diagnosis of IBD. Within 30 years after the diagnosis of IBD, about half of patients experience at least 1 EIM [1]. It has been reported that as many as 25% of patients experience multiple EIMs [9].

Recent studies in Korea have shown a significant increase in the prevalence of IBD compared to the past [10]. Nonetheless, the 5-year survival rate of IBD was found to be 96.6%, which is similar to the rate reported in previous studies in Korea and Japan, and higher than the rates reported for Western countries [11–13]. Since EIMs have a major impact on the quality of life of IBD patients, it is necessary to establish the prevalence of EIMs in IBD patients in Korea, considering the recent increase in IBD. However, epidemiological data regarding EIMs in Korean IBD patients are scarce, and nationwide data for EIMs in Korea have not been obtained. The purpose of this study was to analyze the prevalence of EIMs in Korean IBD patients using national health insurance data.

## Materials and methods

This cross-sectional study was conducted to evaluate the prevalence of EIMs in IBD patients in comparison to the corresponding rates in the general population of Korea.

### Data source

We used the 2014 National Health Insurance Database (NHID) of Korea. The National Health Insurance system is a mandatory health insurance program that covers all Korean residents, so the NHID can play a role as infrastructure for epidemiological studies representing the whole population [14]. The data resource profile of NHID has been published elsewhere [15]. The NHID can be used after approval by the review committee of the National Health Insurance Corporation, and it is accessed in a dedicated data analysis room located within the National Health Insurance Corporation building. Details of this process and a guide for accessing the data are available at http://nhiss.nhis.or.kr/bd/ab/bdaba032eng.do.

The prevalence of EIMs in the general population of Korea was measured using the 2014 National Patient Sample data from the Health Insurance Review and Assessment Service (HIRA). The HIRA randomly samples 3% of all Korean healthcare recipients every year, and provides the resulting databases for research purposes [16]. We obtained the National Patient Sample data (HIRA-NPS-2014-0127) after making a request via the website of the Healthcare Big Data Hub (http://opendata.hira.or.kr) operated by the Korean Health Insurance Review and Assessment Service.

The prevalence of EIMs in the general population of Korea was measured using the same International Classification of Diseases, Tenth Edition (ICD-10) codes as those used in IBD patients [17].

### Inclusion criteria of patients and the comparison group

In this study, CD and UC were defined according to the corresponding ICD-10 codes (CD with K50, and UC with K51) and registration codes from the rare/intractable diseases (RID) patient-support program (CD: V130, UC: V131). The RID registration program was established in 2009 in order to lessen the burden on patients who are suffering from these diseases in Korea. In order for these IBD patients to be eligible for benefits, their doctor needs to submit a separate form to prove that the condition has been confirmed. Although the validity of these codes has not been evaluated in a separate publication, it was confirmed through a review of the medical records in a study by Kim et al. [10]. As a denominator to investigate the prevalence of EIM, IBD patients from the NHID 2014 data between January 1, 2014 and December 31, 2014 were defined according to the following criteria: 1) a diagnosis with the corresponding ICD-10 codes (CD: K50, UC: K51) in at least 1 claim, and 2) the presence of a registration code from the RID patient-support program (CD: V130, UC: V131) in at least 1 claim. Subjects who were under 19 years of age or older than 75 years of age were excluded. To calculate the prevalence of EIMs in the general population of Korea, the same criteria were applied in the 2014 National Patient Sample data.

Age, sex, and drug utilization patterns were compared between CD and UC patients. The variables considered in the analysis of drug utilization patterns were anti-inflammatory drugs (corticosteroids, mesalazine, and balsalazide), immunosuppressants (methotrexate, azathioprine, sulfasalazine, and 6-mercaptopurine) and tumor necrosis factor (TNF)-alpha inhibitors (adalimumab, infliximab, golimumab, and etanercept). To define the age and sex of patients, we used the National Health Insurance subscriber information. In the NHID, this information was obtained from the 2014 subscriber information database. The NPS did not have such a database, so the age of patients was determined based on their age recorded in the first claim in 2014. Anti-inflammatory drugs, immunosuppressants, and tumor necrosis factor (TNF)-alpha inhibitors were identified using the NHID formulary, and exposure to the drugs was defined as presence of more than 1 the prescription code between January 1, 2014 and December 31, 2014. EIMs in the analysis were categorized into 4 categories: ophthalmologic EIMs (scleritis, episcleritis, and iridocyclitis), hepatopancreaticobiliary EIMs (cholelithiasis, sclerosing cholangitis, and acute pancreatitis), dermatologic EIMs (aphthous stomatitis, psoriasis, erythema nodosum, pyoderma gangrenosum, and Sweet syndrome), and musculoskeletal EIMs (rheumatoid arthritis, psoriatic arthritis, ankylosing spondylitis, sacroiliitis, osteoporosis, and osteomalacia). The corresponding ICD-10 codes are presented in S1 Table.

### Statistical analysis

The baseline characteristics were summarized using the mean and standard deviation for continuous variables and frequency and proportion for categorical variables. The Student t-test was used for the comparison of continuous variables, and the chi-square test or the Fisher exact test was used for categorical variables as appropriate.

The prevalence of an EIM was calculated as the number of patients with that EIM divided by the number of IBD patients. The prevalence of each EIM was presented for CD and UC patients, respectively, with 95% confidence intervals (CIs). We used indirect standardization to compare EIM prevalence between IBD patients and the general population of Korea [18, 19]. The distribution of EIMs in the general population of Korea by age and sex is presented in S2 Table. To estimate the age/sex-standardized prevalence ratio, the number of expected events of each EIM in CD and UC patients was calculated by multiplying the age/sex-specific EIM prevalence in the general population by the number of patients in each age/sex stratum of CD and UC patients, and then summing the total number of expected events. The age/sex-standardized prevalence ratios with 95% CIs were calculated for each EIM as the number of expected events divided by the number of observed events. All statistical tests were 2-sided, and P values < 0.0038 (0.05/13, where 13 equals the number of tests) were considered to indicate statistical significance based on the Bonferroni correction to cope with multiple comparisons. SAS version 9.4 (SAS Institute, Inc., Cary, NC, USA) was used for the statistical analysis.

### Ethical considerations

This study protocol was exempted from review by the Institutional Review Board of Seoul National University Hospital and the Seoul National University College of Medicine (IRB number: E-1510-071-711).

## Results

A total of 13,925 CD patients and 29,356 UC patients were included in the analysis. The CD patients were younger and more likely to be male than the UC patients. The use of anti-inflammatory drugs was less frequent in the CD patients than in the UC patients, but the use of immunosuppressants was more common, except for sulfasalazine. The use of TNF-alpha inhibitors was more common in the CD patients than the UC patients, and the most common such drugs were adalimumab and infliximab (Table 1).

**Table 1:** Characteristics of patients with Crohn disease and patients with ulcerative colitis.

Characteristics	Crohn disease (n = 13925)	Ulcerative colitis (n = 29356)	*P* value^*a*^
Age, years, mean ± SD	34.0 ± 13.8	45.7 ± 14.4	< .0001
Male sex	8846 (63.53%)	17067 (58.14%)	< .0001
Anti-inflammatory drugs			
Corticosteroids	6701 (48.12%)	16002 (54.51%)	< .0001
Mesalazine	9576 (68.77%)	20641 (70.31%)	0.0011
Balsalazide	160 (1.15%)	3162 (10.77%)	< .0001
Immunosuppressants			
Methotrexate	178 (1.28%)	186 (0.63%)	< .0001
Azathioprine	6462 (46.41%)	3758 (12.8%)	< .0001
Sulfasalazine	414 (2.97%)	2223 (7.57%)	< .0001
6-mercaptopurine	593 (4.26%)	173 (0.59%)	< .0001
TNF-alpha inhibitors			
Adalimumab	865 (6.21%)	322 (1.1%)	< .0001
Infliximab	2056 (14.76%)	971 (3.31%)	< .0001
Golimumab	4 (0.03%)	6 (0.02%)	*0*.*7361*^*b*^
Etanercept	5 (0.04%)	14 (0.05%)	*0*.*8065*^*b*^

SD, standard deviation; TNF, tumor necrosis factor.

^a^ P values were calculated using the chi-square test.

^b^ P values were calculated using the Fisher exact test.

Among the 17 EIMs investigated, pyoderma gangrenosum, osteomalacia, Sweet syndrome, and scleritis were observed in very few patients. We did not find any female patients with Sweet syndrome. No female CD patients showed scleritis. Ankylosing spondylitis, aphthous stomatitis, acute pancreatitis, cholelithiasis, rheumatoid arthritis, and osteoporosis were found in more than 10 patients per 1000 CD cases, while the prevalence rates of psoriasis, aphthous stomatitis, acute pancreatitis, cholelithiasis, rheumatoid arthritis, osteoporosis, and iridocyclitis in UC patients were more than 10 patients per 1000 cases (Table 2, Table 3).

**Table 2:** Number of individuals with extraintestinal manifestations among Crohn disease patients, ulcerative colitis patients, and the general population.

Extraintestinal manifestations	Crohn disease			Ulcerative colitis			Random sample of Korean population		
	All	Male	Female	All	Male	Female	All	Male	Female
	n = 13,925	n = 9,761	n = 4,164	n = 29,356	n = 16,920	n = 12,436	n = 1,127,261	n = 545,854	n = 581,407
Ophthalmologic EIMs									
Scleritis	6(0.0)	6(0.1)	0(0.0)	29(0.1)	20(0.1)	9(0.1)	491(0.0)	200(0.0)	291(0.1)
Episcleritis	55(0.4)	37(0.4)	18(0.4)	139(0.5)	77(0.5)	62(0.5)	4051(0.4)	1689(0.3)	2362(0.4)
Iridocyclitis	107(0.8)	77(0.8)	30(0.7)	339(1.2)	189(1.1)	150(1.2)	5524(0.5)	2842(0.5)	2682(0.5)
Hepatopancreaticobiliary EIMs									
Cholelithiasis	257(1.8)	176(1.8)	81(1.9)	387(1.3)	234(1.4)	153(1.2)	8117(0.7)	4314(0.8)	3803(0.7)
Sclerosing cholangitis	33(0.2)	20(0.2)	13(0.3)	96(0.3)	52(0.3)	44(0.4)	638(0.1)	379(0.1)	259(0.0)
Acute pancreatitis	439(3.2)	297(3)	142(3.4)	600(2)	351(2.1)	249(2)	8846(0.8)	4520(0.8)	4326(0.7)
Dermatologic EIMs									
Aphthous stomatitis	508(3.6)	301(3.1)	207(5)	1088(3.7)	588(3.5)	500(4)	39824(3.5)	16353(3)	23471(4)
Psoriasis	123(0.9)	83(0.9)	40(1)	359(1.2)	219(1.3)	140(1.1)	8413(0.7)	4693(0.9)	3720(0.6)
Erythema nodosum	32(0.2)	10(0.1)	22(0.5)	34(0.1)	13(0.1)	21(0.2)	341(0.0)	95(0.0)	246(0.0)
Pyoderma gangrenosum	8(0.1)	5(0.1)	3(0.1)	21(0.1)	10(0.1)	11(0.1)	173(0.0)	93(0.0)	80(0.0)
Sweet syndrome	3(0.0)	3(0.0)	0(0.0)	4(0.0)	4(0.0)	0(0.0)	18(0.0)	7(0.0)	11(0.0)
Musculoskeletal EIMs									
Rheumatoid arthritis	496(3.6)	250(2.6)	246(5.9)	1180(4)	476(2.8)	704(5.7)	27648(2.5)	9007(1.7)	18641(3.2)
Psoriatic arthritis	69(0.5)	37(0.4)	32(0.8)	125(0.4)	57(0.3)	68(0.5)	603(0.1)	229(0.0)	374(0.1)
Ankylosing spondylitis	161(1.2)	121(1.2)	40(1)	285(1)	175(1)	110(0.9)	2365(0.2)	1413(0.3)	952(0.2)
Sacroiliitis	16(0.1)	10(0.1)	6(0.1)	29(0.1)	15(0.1)	14(0.1)	563(0.0)	194(0.0)	369(0.1)
Osteoporosis	882(6.3)	350(3.6)	532(12.8)	2445(8.3)	626(3.7)	1819(14.6)	64482(5.7)	7206(1.3)	57276(9.9)
Osteomalacia	6(0.0)	4(0.0)	2(0.0)	20(0.1)	5(0.0)	15(0.1)	757(0.1)	101(0.0)	656(0.1)

EIMs, extraintestinal manifestations.

**Table 3:** Number of patients with extraintestinal manifestations per 1000 patients with Crohn disease or ulcerative colitis and 95% confidence intervals.

Extraintestinal manifestations	Crohn disease			Ulcerative colitis		
	All	Male	Female	All	Male	Female
Ophthalmologic EIMs						
Scleritis	0.43 (0.09–0.78)	0.61 (0.12–1.11)	0.00 (0.00–0.00)	0.99 (0.63–1.35)	1.18 (0.66–1.70)	0.72 (0.25–1.20)
Episcleritis	3.95 (2.91–4.99)	3.79 (2.57–5.01)	4.32 (2.33–6.32)	4.73 (3.95–5.52)	4.55 (3.54–5.56)	4.99 (3.75–6.22)
Iridocyclitis	7.68 (6.23–9.13)	7.89 (6.13–9.64)	7.20 (4.64–9.77)	11.55 (10.33–12.77)	11.17 (9.59–12.75)	12.06 (10.14–13.98)
Hepatopancreaticobiliary EIMs						
Cholelithiasis	18.46 (16.22–20.69)	18.03 (15.39–20.67)	19.45 (15.26–23.65)	13.18 (11.88–14.49)	13.83 (12.07–15.59)	12.30 (10.37–14.24)
Sclerosing cholangitis	2.37 (1.56–3.18)	2.05 (1.15–2.95)	3.12 (1.43–4.82)	3.27 (2.62–3.92)	3.07 (2.24–3.91)	3.54 (2.49–4.58)
Acute pancreatitis	31.53 (28.62–34.43)	30.43 (27.02–33.83)	34.10 (28.59–39.61)	20.44 (18.82–22.06)	20.74 (18.60–22.89)	20.02 (17.56–22.48)
Dermatologic EIMs						
Aphthous stomatitis	36.48 (33.37–39.60)	30.84 (27.41–34.27)	49.71 (43.11–56.31)	37.06 (34.90–39.22)	34.75 (31.99–37.51)	40.21 (36.75–43.66)
Psoriasis	8.83 (7.28–10.39)	8.50 (6.68–10.32)	9.61 (6.64–12.57)	12.23 (10.97–13.49)	12.94 (11.24–14.65)	11.26 (9.4–13.11)
Erythema nodosum	2.30 (1.50–3.09)	1.02 (0.39–1.66)	5.28 (3.08–7.49)	1.16 (0.77–1.55)	0.77 (0.35–1.19)	1.69 (0.97–2.41)
Pyoderma gangrenosum	0.57 (0.18–0.97)	0.51 (0.06–0.96)	0.72 (0.00–1.54)	0.72 (0.41–1.02)	0.59 (0.22–0.96)	0.88 (0.36–1.41)
Sweet syndrome	0.22 (0.00–0.46)	0.31 (0.00–0.66)	0.00 (0.00–0.00)	0.14 (0.00–0.27)	0.24 (0.00–0.47)	0.00 (0.00–0.00)
Musculoskeletal EIMs						
Rheumatoid arthritis	35.62 (32.54–38.70)	25.61 (22.48–28.75)	59.08 (51.92–66.24)	40.20 (37.95–42.44)	28.13 (25.64–30.62)	56.61 (52.55–60.67)
Psoriatic arthritis	4.96 (3.79–6.12)	3.79 (2.57–5.01)	7.68 (5.03–10.34)	4.26 (3.51–5.00)	3.37 (2.50–4.24)	5.47 (4.17–6.76)
Ankylosing spondylitis	11.56 (9.79–13.34)	12.40 (10.20–14.59)	9.61 (6.64–12.57)	9.71 (8.59–10.83)	10.34 (8.82–11.87)	8.85 (7.2–10.49)
Sacroiliitis	1.15 (0.59–1.71)	1.02 (0.39–1.66)	1.44 (0.29–2.59)	0.99 (0.63–1.35)	0.89 (0.44–1.33)	1.13 (0.54–1.72)
Osteoporosis	63.34 (59.29–67.38)	35.86 (32.17–39.55)	127.76 (117.62–137.9)	83.29 (80.13–86.45)	37.00 (34.15–39.84)	146.27 (140.06–152.48)
Osteomalacia	0.43 (0.09–0.78)	0.41 (0.01–0.81)	0.48 (-0.19–1.15)	0.68 (0.38–0.98)	0.30 (0.04–0.55)	1.21 (0.60–1.82)

EIMs, extraintestinal manifestations.

The age/sex-standardized prevalence rate ratios, which were calculated using the sample data of the general population of Korea, were greater than 1 for all EIMs (Table 4). However, the 95% CIs of osteomalacia, Sweet syndrome, episcleritis, and scleritis in CD patients, which had a small number of observed events, included unity. Osteomalacia and Sweet syndrome in UC patients also showed standardized prevalence ratios without statistical significance. Aphthous stomatitis, rheumatoid arthritis, and osteoporosis were highly prevalent in both CD and UC patients, but the standardized prevalence rate ratios of the EIMs were not high.

**Table 4:** Age- and sex-standardized prevalence ratios and 95% confidence intervals of the extraintestinal manifestations using a random sample of the entire Korean population as the reference population.

Extraintestinal manifestations	Crohn disease			Ulcerative colitis		
	Observed events	Expected events	Standardized prevalence ratios (95% CI)	Observed events	Expected events	Standardized prevalence ratios (95% CI)
Ophthalmologic EIMs						
Scleritis	6	4.7	1.28 (0.26–2.30)	29	12.9	2.24 (1.43–3.06)
Episcleritis	55	42.2	1.30 (0.96–1.65)	139	105.5	1.32 (1.10–1.54)
Iridocyclitis	107	55.6	1.92 (1.56–2.29)	339	151.7	2.23 (2.00–2.47)
Hepatopancreaticobiliary EIMs						
Cholelithiasis	257	63.4	4.06 (3.56–4.55)	387	233.7	1.66 (1.49–1.82)
Sclerosing cholangitis	33	4.4	7.46 (4.92–10.01)	96	18.9	5.09 (4.07–6.10)
Acute pancreatitis	439	88.9	4.94 (4.47–5.40)	600	242	2.48 (2.28–2.68)
Dermatologic EIMs						
Aphthous stomatitis	508	412.4	1.23 (1.12–1.34)	1,088	1,027.7	1.06 (1.00–1.12)
Psoriasis	123	94.5	1.30 (1.07–1.53)	359	232.2	1.55 (1.39–1.71)
Erythema nodosum	32	3.2	9.91 (6.47–13.34)	34	8.4	4.06 (2.69–5.42)
Pyoderma gangrenosum	8	1.8	4.43 (1.36–7.50)	21	4.8	4.36 (2.50–6.23)
Sweet syndrome	3	0.1	20.60 (0.00–43.91)	4	0.5	8.06 (0.16–15.96)
Musculoskeletal EIMs						
Rheumatoid arthritis	496	217.3	2.28 (2.08–2.48)	1,180	728.8	1.62 (1.53–1.71)
Psoriatic arthritis	69	4.7	14.59 (11.14–18.03)	125	16.3	7.68 (6.34–9.03)
Ankylosing spondylitis	161	30.5	5.28 (4.46–6.10)	285	65.4	4.36 (3.85–4.87)
Sacroiliitis	16	4.7	3.37 (1.72–5.02)	29	14.7	1.98 (1.26–2.70)
Osteoporosis	882	330	2.67 (2.50–2.85)	2,445	1,590.2	1.54 (1.48–1.60)
Osteomalacia	6	4.8	1.26 (0.25–2.26)	20	18.4	1.09 (0.61–1.56)

CI, confidence interval; EIMs, extraintestinal manifestations.

## Discussion

This is the first study to evaluate the prevalence rates of 17 ophthalmologic, hepatopancreaticobiliary, dermatologic, and musculoskeletal EIMs with a cross-sectional design using Korean national health insurance claim data from 2014. Statistically significant higher standardized prevalence rate ratios were observed, except for some EIMs with extremely low frequencies. Among the statistically significant EIMs, the highest standardized morbidity rate ratio was estimated for psoriatic arthritis, followed by erythema nodosum and sclerosing cholangitis in CD, and psoriatic arthritis, sclerosing cholangitis, and ankylosing spondylitis in UC. The standardized prevalence rate ratios were generally higher in CD than in UC.

Age, sex, and medication utilization patterns were significantly different in CD and UC patients, and differences in CD and UC were found for standardized morbidity rate ratios. The medication utilization pattern is likely to be influenced by physician subspecialty. Because musculoskeletal EIMs are the most frequent, it may be expected that there will be differences in the medicines prescribed for patients with IBD depending on whether they are seen by a rheumatologist or a non-rheumatologist. The treatment of severe musculoskeletal EIMs may often involve close collaboration between rheumatologists and gastroenterologists. For example, when choosing an immunomodulatory agent, rheumatologists tend to prefer methotrexate and gastroenterologists tend to prefer azathioprine. Regarding TNF inhibitors, although etanercept has no effect on IBD, as infliximab and adalimumab tend to be preferred by rheumatologists. Rheumatologists tend to prescribe more sulfasalazines, while gastroenterologists prefer to use mesalazine in comparison to rheumatologists.

According to Koutroubakis et al., arthralgia, peripheral arthritis, aphthous stomatitis, and erythema nodosum were the most frequent EIMs among 1860 Greek patients with IBD. EIMs were more prevalent in females and in patients with CD [20]. In a retrospective study using the national health insurance database of Taiwan between 1998 and 2011 including 3153 IBD patients, peripheral arthritis was the most common EIM, followed by ankylosing spondylitis and osteoporosis. The prevalence of EIMs was higher in CD patients and in females [21]. In a population-based study that used the Manitoba Health administrative databases (1984–1996), EIMs were defined based on the presence of at least five relevant claims in IBD patients. The 10-year prevalence rates for iritis/uveitis, primary sclerosing cholangitis (PSC), ankylosing spondylitis, pyoderma gangrenosum, and erythema nodosum using their definition ranged from 0.6% to 2.8%. Compared with the matched general population cohort, the prevalence of EIM was relatively high in IBD patients [5]. As the prevalence of EIMs varies across studies owing to differences in the study periods and the definitions of EIMs, it is difficult to compare study findings directly. However, the tendency for EIMs to be more prevalent in females, CD patients, and IBD patients than in the general population, which was reported in previous studies, was also confirmed in our study.

### Ophthalmologic EIMs

Following joints and the skin, the eye is the site of many immune-related EIMs [22]. According to previous studies conducted in India, Switzerland, and Turkey, ophthalmologic EIMs occur in up to 13% of IBD patients [23–25], and are more frequent in CD patients than in UC patients [26], although our results showed that they tended to be more common in UC patients on a frequency basis. However, this difference was not prominent when the standardized prevalence rate ratio was compared. Rather, the main inconsistency we found with previous studies is that iridocyclitis was more frequently observed than episcleritis. Previous studies have found episcleritis to be the most common ocular EIM, whereas uveitis was relatively rare [7]. In our study, iridocyclitis was more common, but another previous study conducted in Switzerland reported a higher prevalence rate for uveitis than was found in this study [24]. Thus, episcleritis may be less likely to be a comorbidity in Korean IBD patients.

### Hepatopancreaticobiliary EIMs

Up to 50% of IBD patients may have hepatobiliary manifestations [7]. PSC has a well-known association with IBD. Seventy-five percent of PSC patients have UC, and 5%-10% of PSC patients have CD [27, 28]. Moreover, 5% of UC patients and 2% of CD patients develop PSC [29]. Ye et al. reported that the prevalence of PSC was 1.1% among 1849 Korean ulcerative colitis patients using medical records (from July 1977 to September 2009) of a tertiary hospital [30]. In our study, the prevalence of sclerosing cholangitis was one-tenth of the reported prevalence, but a tendency for a higher prevalence in UC patients than in CD patients was observed.

Cholelithiasis is a common EIM in IBD patients, and is frequently seen in cases of CD, especially those involving the ileum. It has been reported that up to 13%-34% of cases are caused by obstruction of the enterohepatic circulation, which is known to occur more frequently in IBD patients than in the general population of Sweden [31]. Our data indicated that this condition occurred more frequently in CD patients than in UC patients, and this tendency was more pronounced in comparison to the general population.

Acute pancreatitis is a common side effect of 6-mercaptopurine or azathioprine treatment. However, it is associated with gallstones and is more common in cases of CD. A retrospective multicenter study in Spain reported that acute pancreatitis occurred in 1.6% of IBD patients [32]. A Dutch study reported that acute pancreatitis was four times more common in CD patients and two times more common in UC patients than in the general population [33], which is very similar to our data.

### Dermatologic EIMs

It has been reported that 2%-34% of IBD patients experience dermatologic EIMs [34]. In a study performed in Turkey, dermatologic EIMs were present in 9.3% of patients; erythema nodosum was present in 7.4% of patients, and pyoderma gangrenosum in 2.3% [35]. Erythema nodosum has been reported in 15% of CD patients and in 10% of UC patients [36], and it has been reported to be more common in women [36]. Our study also confirmed the tendency for this condition to be more common in CD patients and for there to be a higher prevalence in women. Pyoderma gangrenosum is a much rarer, more severe, and debilitating EIM that is more common in UC patients than in CD patients [37–39]. Its prevalence has been reported to be 1%-10% in UC patients and 0.5%-20% in CD patients [40].

Psoriasis is more common in CD patients, and is known to be more common in CD than in the general population of the United States [41]. Although the prevalence rates identified in our study were lower, the standardized prevalence rate ratio was higher in UC patients than in CD patients.

Oral lesions including aphthous stomatitis are known to be common in IBD patients, and are more common in CD patients [9, 22, 42]. In our data, the prevalence rate was 3%-4% in both CD and UC patients; this rate was significantly higher than that observed in the general population of Korea, but the magnitude of the difference was not large.

Acute febrile neutrophilic dermatosis (Sweet syndrome) is a rare dermatologic EIM [43–45]. The literature on its prevalence is scarce, and only case reports are available. Our data differ from previous reports in that it has been reported to be more common in women, but there were no female patients with this condition in our data. However, there were not enough cases in our data to assess the prevalence rate in a meaningful way.

### Musculoskeletal EIMs

Traditionally, the main musculoskeletal EIM is seronegative arthralgia/arthritis [46]. Approximately 5%-10% of UC patients and 10%-20% of CD patients experience these symptoms [47]. Axial arthropathies are less common than peripheral arthralgia/arthritis, and have been reported to be experienced by 3% to 5% of IBD patients [22, 26, 48]. Asymptomatic sacroiliitis can be observed in radiological examinations in up to 52% of CD patients [49]. Although ankylosing spondylitis and isolated sacroiliitis are distinct diseases, sacroiliitis can be reported as ankylosing spondylitis in the health insurance claims data, so it is possible that the prevalence of sacroiliitis was underestimated and that of ankylosing spondylitis was overestimated. When ankylosing spondylitis and sacroiliitis were grouped as axial arthropathy and rheumatoid arthritis and psoriatic arthritis were grouped as peripheral arthralgia/arthritis, the trends reported in the existing literature were reproduced in our study.

Patients with IBD have a high risk of osteoporosis, with consequences including corticosteroid therapy, decreased physical activity, inflammation-related bone resorption, and dietary malabsorption of minerals [50]. As a result, patients with IBD have a greater risk of fractures than the general population of Korea, and this risk is higher in both men and women. The prevalence of osteoporosis has been found to be 7%-35% in CD patients [51–53] and 18% in UC patients [52]. In our data, the prevalence rate was higher in women and the standardized prevalence rate was higher in CD patients.

This study has the advantage of high representativeness because it used health insurance claim data including all Koreans. Due to some limitations, however, some precautions should be taken regarding the correct interpretation of the results. First, the validity of the diagnostic codes for EIMs should be carefully considered. There is a possibility that the prevalence rate was underestimated due to the low claim rate in routine clinical practice for erythema nodosum, pyoderma gangrenosum, and osteomalacia, while overestimation of EIMs could have occurred due to the inclusiveness of the ICD-10 codes for rheumatoid arthritis and iridocyclitis. For the comparison with the general population, however, there is little concern about underestimation and overestimation because the prevalence was measured in the same way in both databases.

Even though we calculated standardized prevalence ratios by adjusting for age and sex, other possible confounding variables were not considered. In particular, obesity has recently been reported to be associated with EIMs [54]; however, it was not possible to evaluate whether patients were obese using the claims database, because this information was not included. Moreover, no further examination was conducted of the relationships between drug utilization patterns and EIM prevalence in this study. Due to the limitation of the cross-sectional study design for evaluating drug effects, we suggest that the associations between EIMs and drug utilization should be carefully interpreted with evidence from studies with higher on the evidence hierarchy. A cohort study with a new-user design would be needed to provide relevant evidence, since the rarity of both IBD and some EIMs will limit the availability of appropriate patients for randomized controlled trials.

This study presents data on the prevalence of each EIM in IBD patients. Considering the possible impact of EIMs on the quality of life of the patients, the high prevalence presented in this study suggests that more attention and appropriate treatment are needed. The study also confirmed that the EIMs are more prevalent in Korean IBD patients than in the general population of Korea, which suggests that more attention should be paid to EIMs in IBD patients in everyday clinical practice, and that our results can be used for patient education.

## Supporting information

S1 Table Extraintestinal manifestations and ICD-10 codes included in the analysis. Abbreviations: EIM, extraintestinal manifestation; ICD-10, International Classification of Diseases, Tenth Edition.
(DOCX)

S2 Table Distribution of EIMs in the general population by age and sex. Abbreviations: EIM, extraintestinal manifestation.
(DOCX)
