# Supplementary material for: Prevalence of extraintestinal manifestations in Korean inflammatory bowel disease patients
Source: PLoS One. 2018 Jul 10;13(7):e0200363. doi: 10.1371/journal.pone.0200363 (PMC6039042; doi:10.1371/journal.pone.0200363)
Supplement: S1 Table — Abbreviations: EIM, extraintestinal manifestation; ICD-10, International Classification of Diseases, Tenth Edition. (DOCX) [file pone.0200363.s001.docx]

* Supplementary Table 1. Extraintestinal manifestations and ICD-10 codes included in the analysis

| Extraintestinal Manifestations | | ICD-10 Codes |
| --- | --- | --- |
| Ophthalmologic EIMs | Scleritis | H15.0 |
|  | Episcleritis | H15.1 |
|  | Iridocyclitis | H20 |
| Hepatopancreaticobiliary EIMs | Cholelithiasis | K80 |
|  | Sclerosing cholangitis | K83.0 |
|  | Acute pancreatitis | K85 |
| Dermatologic EIMs | Aphthous stomatitis | K12 |
|  | Psoriasis | L40 |
|  | Erythema nodosum | L52 |
|  | Pyoderma gangrenosum | L88 |
|  | Sweet’s syndrome | L98.2 |
| Musculoskeletal EIMs | Rheumatoid arthritis | M05,M06 |
|  | Psoriatic arthritis | M07 |
|  | Ankylosing spondylitis | M45 |
|  | Sacroiliitis | M46.1 |
|  | Osteoporosis | M80,M81 |
|  | Osteomalacia | M83 |

EIMs, extraintestinal manifestations; ICD-10, International Classification of Diseases, Tenth Edition
