# Supplementary material for: Prevalence of extraintestinal manifestations in Korean inflammatory bowel disease patients
Source: PLoS One. 2018 Jul 10;13(7):e0200363. doi: 10.1371/journal.pone.0200363 (PMC6039042; doi:10.1371/journal.pone.0200363)
Supplement: S2 Table — Abbreviations: EIM, extraintestinal manifestation. (DOCX) [file pone.0200363.s002.docx]

S2 Table. Distribution of EIMs in the general population by age and sex

| Sex | Male | | | | | | | Female | | | | | | |
| --- | --- | --- | --- | --- | --- | --- | --- | --- | --- | --- | --- | --- | --- | --- |
| Age group | < 20 | 20-29 | 30-39 | 40-49 | 50-59 | 60-69 | ≥70 | < 20 | 20-29 | 30-39 | 40-49 | 50-59 | 60-69 | ≥70 |
| Number of patients | 18,355 | 88,890 | 106,564 | 118,899 | 112,046 | 63,010 | 38,090 | 18,314 | 91,177 | 111,122 | 123,254 | 116,423 | 68,509 | 52,608 |
| Ophthalmologic EIMs | |  |  |  |  |  |  |  |  |  |  |  |  |  |
| Scleritis | 3 | 19 | 34 | 39 | 44 | 37 | 24 | 5 | 32 | 34 | 37 | 70 | 62 | 51 |
| Episcleritis | 35 | 168 | 335 | 387 | 388 | 239 | 137 | 64 | 296 | 412 | 514 | 557 | 320 | 199 |
| Iridocyclitis | 29 | 251 | 450 | 586 | 641 | 476 | 409 | 44 | 232 | 359 | 385 | 500 | 505 | 657 |
| Hepatopancreaticobiliary EIMs | |  |  |  |  |  |  |  |  |  |  |  |  |  |
| Cholelithiasis | 11 | 112 | 380 | 785 | 1,104 | 1,067 | 855 | 17 | 160 | 399 | 608 | 933 | 838 | 848 |
| Sclerosing cholangitis | 0 | 7 | 21 | 47 | 76 | 110 | 118 | 3 | 5 | 17 | 19 | 44 | 65 | 106 |
| Acute pancreatitis | 76 | 385 | 609 | 909 | 1,153 | 796 | 592 | 88 | 548 | 647 | 770 | 959 | 653 | 661 |
| Dermatologic EIMs | |  |  |  |  |  |  |  |  |  |  |  |  |  |
| Aphthous stomatitis | 454 | 2,048 | 2,688 | 2,959 | 3,362 | 2,703 | 2,139 | 589 | 3,242 | 3,908 | 4,076 | 4,807 | 3,334 | 3,515 |
| Psoriasis | 86 | 494 | 761 | 966 | 1,103 | 790 | 493 | 80 | 498 | 700 | 751 | 811 | 491 | 389 |
| Erythema nodosum | 2 | 15 | 17 | 13 | 20 | 17 | 11 | 7 | 36 | 34 | 51 | 68 | 31 | 19 |
| Pyoderma gangrenosum | 2 | 9 | 13 | 18 | 16 | 20 | 15 | 1 | 8 | 13 | 22 | 17 | 14 | 5 |
| Sweet syndrome | 0 | 1 | 0 | 1 | 2 | 1 | 2 | 0 | 0 | 2 | 1 | 3 | 5 | 0 |
| Musculoskeletal EIMs | |  |  |  |  |  |  |  |  |  |  |  |  |  |
| Rheumatoid arthritis | 95 | 671 | 1,172 | 1,675 | 2,271 | 1,838 | 1,285 | 90 | 750 | 1,582 | 3,319 | 5,499 | 4,060 | 3,341 |
| Psoriatic arthritis | 0 | 19 | 25 | 34 | 62 | 43 | 46 | 0 | 17 | 16 | 51 | 97 | 101 | 92 |
| Ankylosing spondylitis | 32 | 183 | 359 | 281 | 265 | 185 | 108 | 8 | 83 | 143 | 180 | 215 | 145 | 178 |
| Sacroiliitis | 4 | 14 | 31 | 29 | 52 | 33 | 31 | 4 | 31 | 36 | 60 | 103 | 78 | 57 |
| Osteoporosis | 17 | 80 | 220 | 506 | 1,294 | 1,947 | 3,142 | 22 | 228 | 856 | 3,420 | 14,053 | 19,045 | 19,652 |
| Osteomalacia | 1 | 2 | 9 | 15 | 21 | 21 | 32 | 2 | 46 | 69 | 60 | 183 | 178 | 118 |

EIMs, extraintestinal manifestations
